# Physicochemical and Bioactivity Characteristics of Doxycycline Hyclate-Loaded Solvent Removal-Induced Ibuprofen-Based In Situ Forming Gel

**DOI:** 10.3390/gels9020128

**Published:** 2023-02-03

**Authors:** Napaphol Puyathorn, Setthapong Senarat, Nutdanai Lertsuphotvanit, Thawatchai Phaechamud

**Affiliations:** 1 Programme of Pharmaceutical Engineering, Faculty of Pharmacy, Silpakorn University, Nakhon Pathom 73000, Thailand; 2 Program of Pharmaceutical Technology, Department of Pharmaceutical Technology, Faculty of Pharmacy, Silpakorn University, Nakhon Pathom 73000, Thailand; 3 Department of Industrial Pharmacy, Faculty of Pharmacy, Silpakorn University, Nakhon Pathom 73000, Thailand; 4 Natural Bioactive and Material for Health Promotion and Drug Delivery System Group (NBM), Faculty of Pharmacy, Silpakorn University, Nakhon Pathom 73000, Thailand

**Keywords:** ibuprofen, in situ forming gel, doxycycline hyclate, solvent removal, physicochemical, bioactivity

## Abstract

Modulation with the suppression of infection and inflammation is essential to the successful treatment of periodontitis. An aqueous insoluble hydrophobic anti-inflammatory compound, i.e., ibuprofen (IBU), was investigated in this study as the matrix-forming agent of a doxycycline hyclate (DH)-loaded solvent removal-induced in situ forming gel (ISG) using dimethyl sulfoxide (DMSO) and *N*-methyl pyrrolidone (NMP) as the solvents. Their physicochemical properties, including pH, density, viscosity, surface tension, contact angle, water tolerance, injectability, mechanical properties, gel formation, and drug release, were determined. Their antimicrobial activities were tested using agar cup diffusion, and their anti-inflammatory activity was assessed using thermal inhibition of protein denaturation of egg albumin. Increasing the IBU content decreased the density, pH, surface tension, and contact angle but increased the viscosity, force and work of injection, and gel formation of IBU-based ISG solution. Although their water tolerance values decreased with the increase in IBU content, the addition of DH and the use of NMP led to high water tolerance. The characterization of the dried gel remnants of ISGs presented no change in IBU crystallinity and thermal properties and confirmed no chemical interaction among the components of ISGs. The obtained transformed IBU matrix prolonged the release of DH and IBU from ISGs over 7 days from its tortuously packed IBU matrix with small pores, and conformed well with Fickian diffusion mechanism. The developed DH-loaded solvent removal-induced IBU-based ISGs exhibited efficient antimicrobial activities against *Staphylococcus aureus*, methicillin-resistant *S. aureus*, *Escherichia coli*, *Candida albicans*, *Porphyromonas gingivalis*, and *Aggregatibacter actinomycetemcomitans*. IBU in formulation promoted the antimicrobial activity of ISGs, whereas DH and NMP promoted the anti-inflammatory activity of ISGs. Consequently, the DH-loaded solvent removal-induced IBU-based ISGs proposed in this study show great potential as an effective bioactive drug delivery system for periodontitis treatment by localized periodontal pocket injection.

## 1. Introduction

The Global Burden of Disease Study conducted in 2016 indicated that periodontal disease was the 11th most prevalent condition in the world [1] with a prevalence ranging from 20% to 50% [2]. The bacterial biofilm that forms on the tooth surfaces and the inflammation in the periodontal pocket have been recognized as the main causes of periodontitis [3,4,5,6,7,8]. Because of restricted access to reach the periodontopathic organisms present in the deep periodontal pocket and infected surrounding tissue, which are naturally obtrusive, antibacterial therapy has been used as an adjuvant for conventional mechanical grafting [9,10]. To overcome the cons of systemic antimicrobial administration, the local administration of antimicrobial therapy has attracted prodigious attention [11]. Conventional drug delivery has its share of shortcomings, particularly the rapid and relatively short duration of release of therapeutic drug concentrations at the target site. Local antimicrobial therapy is one such technique that involves the delivery of therapeutic agents directly into the diseased periodontal pocket region with the rationale to deliver and maintain the adequate therapeutic dose of the drug for a sustained period while minimizing its side effects.

The solvent removal-induced in situ forming gel (ISG) system has emerged as an attractive injectable drug delivery system for periodontal pocket delivery because of its ease of administration and a less complicated fabrication process based on the presence of a matrix-forming agent to modulate the drug release [12,13]. This dosage form initially consists of a drug-loaded liquid solution that thereafter solidifies via water–solvent exchange into a drug-entrapped gel-like or solid matrix depot after injection and exposure to the aqueous crevicular fluids [14,15,16]. Atridox^®^ is a commercial product containing doxycycline hyclate (DH) using poly(dl-lactide) as the polymer dissolved in *N*-methyl pyrrolidone (NMP) prepared as the ISG for the controlled release of DH for 7 days [17,18]. Furthermore, Atrisorb-D^®^ FreeFlow™ with DH loading is licensed for local bacterial growth inhibition and healing in a controlled release manner for 7 days [19]. Nevertheless, these products are composed of rather expensive matrix forming agents; therefore, the search for a new material should be conducted. DH is soluble >76.9 µg/mL in water and acts as a hydrophilic antimicrobial compound (Log*P* = −0.25). DH is a bacteriostatic drug that binds reversibly to the 30S ribosomal subunit, as well as the 50S subunit, preventing aminoacyl-tRNA from attaching to the mRNA–ribosome complex, which leads to the inhibition of protein synthesis [18,19]. As in previous studies, ISG formulations containing DH as the model drug were designed for periodontal pocket delivery employing various types of polymers, such as Eudragit RS, ethyl cellulose, and bleached shellac as the matrix-forming agents [20,21,22,23]. DH released from these different polymer-based ISGs containing 20% ethyl cellulose, 40% Eudragit RS, 30% bleached shellac, 10% cholesterol, and 10% benzyl benzoate for 9 h, 16 h, 2 days, and 10 days [20,21,22,23,24,25]. Nevertheless, these drug-loaded polymeric formulations were rather difficult to inject into the target site because of their viscous property. Saturated fatty acids have been recently applied as the matrix-forming agents of ISG for controlling vancomycin hydrochloride for periodontitis treatment [26,27,28]. The less viscous ISG prepared from these short-chain hydrocarbon fatty acids and their apparent hydrophobicity promoted the ease of injection with sustainable drug release.

Ibuprofen (IBU) is a nonsteroidal anti-inflammatory drug (NSAID) with good permeability [29]. IBU is the most common over-the-counter drug for mild to moderate pain and inflammation in certain conditions, including dental pain [30,31] since it reduces the sign and severity of gingivitis [32,33]. IBU-loaded drug delivery systems for local administration in the oral cavity were fabricated for controlled release rate and drug delivery to the target site, such as the in situ gel-forming system [34,35] and hydrogel system (Carbopol^®^) [36]. Furthermore, chlorhexidine–ibuprofen-loaded in situ gel-forming system using poly(lactic-co-glycolic acid) as the polymer presented antibacterial activity against periodontal pathogens, such as *Porphyromonas gingivalis* [34]. In recent years, the formulation concept of “using active chemicals as excipients” has been the focus of considerable interest. This concept applies the physical and chemical properties of active ingredients to assist the preparation or enhance the efficacy of the main active pharmaceutical ingredient. The solubility profile of IBU shows that it has hydrophobic properties and dissolves in organic solvents, such as dimethyl sulfoxide (DMSO) and NMP; thus, this drug can be a carrier of antimicrobial agents, such as DH, to improve the efficacy of periodontitis treatment. The conceptual dual drug delivery system is also interesting for the improvement of the treatment of various diseases [37,38,39]. The dual drug delivery for periodontitis has also been reported by several investigators [34,40,41]; nevertheless, studies on the use of drugs, such as IBU, as the matrix-forming agent of ISG have not been reported. In addition, the less viscous ISG prepared from IBU and its apparent hydrophobicity should be beneficial for ease of injection and prolongation of drug release. Therefore, this research aims to develop DH-loaded solvent removal-induced IBU-based ISG for periodontal delivery. Their physicochemical properties and bioactivities, including antimicrobial and anti-inflammatory activities, were investigated.

## 2. Results and Discussion

### 2.1. Physicochemical Properties

#### 2.1.1. Physical Appearance

DH and IBU were dissolved in DMSO and NMP in a short time during preparation because both drugs could freely solubilize in these aprotic solvents. IBU contents of more than 40% *w*/*w* hardly dissolved in these solvents and some precipitates of excess drug particles appeared. The successful formulation of IBU-based ISGs was indicated by clear, colorless, and low-viscosity solutions. Increasing the IBU content in ISG solutions did not change that visual appearance. The addition of DH to IBU-based ISGs only turned the colorless solution to a clear yellowish solution because DH has a yellowish color.

#### 2.1.2. Density, pH, and Viscosity

The density values of NMP and DMSO were 1.0265 ± 0.0008 and 1.0935 ± 0.0007 g/cm^3^, respectively. The addition of IBU decreased the density of the DMSO series (i.e., from 1.0845 g/cm^3^ to 1.0586 g/cm^3^) and NMP series (i.e., from 1.0264 g/cm^3^ to 1.0245 g/cm^3^, as shown in Table 1) because the density of IBU was 1.029 less than that of DMSO and close to that of NMP [42]. The hydrophobicity and low density of IBU decreased the density of the formulations. Furthermore, the obtained density values were used as the crucial parameter for the subsequent determination and calculation of the surface tension. The addition of DH increased the density of ISG to 1.3809 g/cm^3^ [43].

The pH of pharmaceutical formulations was checked to define the irritation of the formulations to the target site. The pH values of organic solvents, IBU-based ISGs, and DH-loaded ISGs are shown in Table 1. The pH values of DMSO and NMP were significantly higher than those of the formulations (*p* < 0.01), and their pH decreased gradually after subsequently increasing the IBU content because IBU is a propionic acid NSAID and its COOH part donated a proton to the formulation [29,31]. Moreover, the addition of DH significantly decreased the pH of both formulations because of its HCl from hyclate salt [20,21,23]. The pH values of DID40 and DIN40 were 4.93 ± 0.16 and 5.04 ± 0.08, respectively. Therefore, their pH values were acceptable because they were close to the pH of 6.8 of periodontal pocket tissue and typically a tiny amount of dosage form is applied during administration to periodontal pocket.

The viscosity of IBU-loaded ISGs slightly increased with the increase in IBU content, as presented in Table 1. ID40 and IN40 had significantly higher values than the other formulations (*p* < 0.05). The intermolecular interaction between the COOH part of IBU, the S=O part of DMSO, and the C=O part of NMP should be H-bonding that diminished the solvent molecular movement. The addition of DH to the formulation also significantly increased the viscosity of the systems, which was significantly higher than that of the DH-free formulation (*p* < 0.05) because of the steric hindrance characteristic of the DH structure obstructing solvent molecular movement [22,23]. Moreover, the decrease in solvent content resulting from the incorporation of IBU and DH enhanced the solute concentration and environmental viscosity. By comparison, the viscosities of the DMSO and NMP series were quite similar. The viscosities of ID40, IN40, DID40, and DIN40 were 12.28 ± 0.07, 13.86 ± 0.10, 18.61 ± 0.19, and 20.82 ± 0.37 cP, respectively. However, DID40 and DIN40 presented remarkably low viscosity (<20 cP) compared with polymer-based ISG because of the low density, small size, and unsophisticated structure of loaded IBU [29,30]. By comparison, the viscosities of the IBU-loaded ISGs were significantly lower than other polymer-based ISGs from previous works. For example, the viscosities of 20% *w*/*w* ethyl cellulose, 30% *w*/*w* bleach shellac, and 35% *w*/*w* Eudragit RS-based ISG were 2200, 120, and 300 cPs, respectively. Furthermore, the ISGs containing 25% *w*/*w* ethyl cellulose, 35% *w*/*w* bleach shellac, and 40% Eudragit RS could not flow because of their too-viscous features [25,44]. For natural resin-based ISG, the viscosities of 25% to 50% *w*/*w* benzoin in NMP and DMSO were 6.61–171.46 and 19.77–137.03 cP, respectively, compared with propolis (i.e., 119–155 and 80.96–577.90 cP, respectively) [16]. The benefit of the low-viscosity IBU-based ISG was the simplicity of preparation by conventional mixing technique and ease of administration via injection similar to the ISGs prepared using saturated fatty acids [27,28] or borneol [15] as the matrix-forming agents.

#### 2.1.3. Surface Tension and Contact Angle

Normally, the surface tension at the fluid–air interfaces is attributed to the greater attraction of liquid molecules to each other because of cohesive force promoting the tendency of minimum liquid surfaces. The surface tension of the binary mixture of IBU-based and drug-loaded ISGs has not been reported previously. Table 1 shows the surface tensions of solvent, IBU-based ISG, and DH-loaded IBU-based ISG. The surface tensions of NMP and DMSO were 39.31 ± 0.28 and 43.95 ± 0.13 mN/m, respectively. With the addition of 10% to 40% *w*/*w* IBU, the surface tensions of DMSO and NMP decreased from 40.18 ± 0.41 to 35.10 ± 0.87 and from 39.42 ± 0.19 to 36.91 ± 0.24, respectively. Moreover, the addition of DH to the ISG formulation decreased the surface tension. This reduction of surface tension due to the attractive or cohesive force among solvent molecules around the surface region occurred through the interference of dissolved IBU and DH molecules. Both DMSO and NMP are polar aprotic solvents with strong dipole force and hydrogen bonding interactions between their molecules, whereas IBU is a monocarboxylic acid presenting a steric hindrance effect [45,46,47]. Thus, DMSO and NMP have a higher bonding strength between their molecules than IBU. For a binary mixture, the component with the strongest molecular interaction typically simmers down in the bulk fluid phase instead of interacting on the droplet surface. Practically, the compound having the lowest molecular interaction is normally expelled from the bulk fluid to the droplet surface because of the apparent attractive forces between solvent molecules [48,49,50]. This behavior of diminished surface tension might be beneficial to the administration of the formulation and delivery of DH to the target membrane for treatment because the low surface tension enhanced their spreadability on the tissue surface due to the decreased cohesive force of the liquid droplet.

The contact angle of ISGs directly represented the wettability or spreadability of the formulation on the target surface. The incorporation of IBU tended to reduce the contact angle of the formulation using DMSO or NMP as solvents on both glass slide and paraffin surfaces, signifying the increased hydrophobicity of the formula because the addition of IBU promoted more prominent adhesion on both glass slide and paraffin surfaces. The contact angles on both the glass slide and glass slide covered with paraffin decreased (*p* < 0.05; compared with their solvents) in an IBU concentration-dependent manner, representing the strong hydrophobic interaction between IBU molecules (Log *K*_o/w_ of 3.97) and the hydrophobic surface of paraffin [29]. Nevertheless, the rather high contact angle tended to be evident at 30% and 40% IBU because their high viscosities retarded the spreadability of the droplet on these two surfaces [51]. The agarose surface prepared with PBS pH 6.8 was fabricated for demonstration as a periodontal pocket-like surface, because it is composed of ion species dissolved in water as the main component in the gelling state. Its smooth planar topography was also a suitable surface for the measurement of contact angle. Notably, the contact angle of IBU-loaded ISGs was significantly higher on the agarose surface than those of pure organic solvents, such as DMSO and NMP, because of the in situ transformation from solution into solid-like matrix, which provokes the solvent exchange mechanism between organic solvent in the formulation and aqueous phase in agarose gels [15]. These obtained matrices from solvent removal at the contact interface between the formulation droplet and agar decreased the spreadability of the initial IBU-loaded ISG droplet [15]. The apparent minimal contact angles of NMP and DMSO were observed, and their values were significantly less than those of the formulations (*p* < 0.01; Table 1). Nevertheless, the contact angle decreases with the increase in IBU concentration or the addition of DH to the ISG system (Table 1). This result could be attributed to the fact that the incorporated drugs also reduced the strength of the interaction between molecules similar to that of surface tension, as described previously; thereafter, the extent of spreading increased, whereas the contact angle decreased, indicating the difficulty in matrix formation. Although their contact angle values were rather high, they remained less than 90°, signifying their good wettability on the target surfaces [52]. The wettability of ISG is required for the adhesive quality between the formulation and the target surface, so as to prevent the undesirable slipping out of the drug-loaded IBU matrix from the periodontal pocket [46,53].

#### 2.1.4. Water Tolerance

The water tolerance value was the crucial design parameter that indicated the amount of aqueous phase used to induce the phase separation of solvent removal-induced ISG systems. Therefore, the capacity of solution formulation to endure a phase change induced by the aqueous phase was compared with the water tolerance value. In this research, the amount of water required to achieve phase separation was used to indicate the water tolerance property of the prepared IBU-based ISG systems. Therefore, the need for a larger amount of water to obtain turbidity (endpoint) indicates a higher water tolerance [27,28]. The amount of water needed to achieve the phase separation point or water tolerance value of DH-loaded IBU-based ISGs is presented in Table 2. With the increase in IBU content in ISGs, the water tolerance notably showed a tendency to decrease, signifying sensitivity to the aqueous phase. At higher IBU concentrations, the solvent was insufficient to dissolve the drug whenever the solvent diffused out. The water tolerance of ISGs also decreased after the addition of DH to the formulation. Both the increase in IBU concentration and addition of DH enhanced the total solute molecules and diminished the solvent component in the system; therefore, IBU reached the saturated dissolving point easier because of its low hydrophilicity. Moreover, the gradual addition of water into the ISG system reduced the Log*P* of the system because of the higher dielectric constant of water than the organic solvent of ISGs. The dielectric constant values of DMSO, NMP, and water are 47, 32, and 78.54, respectively, at 20 °C [54,55]. Practically, hydrophobic substances, such as IBU, are crystallized by the Log*P* reduction phenomenon. ISGs of the DMSO series presented a lower water tolerance than the NMP series because the Log*P* of DMSO (−1.35) is lower than that of NMP (−0.38) [56], which induced the crystallization of IBU to crystallize and the easier phase separation from the DMSO solution than from the NMP solution. The decreasing water tolerance of saturated fatty acid-based ISG in DMSO was evident as the carbon chain of fatty acids was longer or their concentrations were higher [27,28]. Lauric acid-based ISG in DMSO systems required less water than the systems using NMP as a solvent to achieve the endpoint because DMSO has higher water miscibility than NMP because of its higher polarity [57], leading to the fast phase separation of lauric acid from DMSO. The increasing temperature of the system from room temperature to body temperature could increase the water tolerance of all formulas (Table 2) because the ideal solubility of a crystalline solute in a liquid solvent is increased proportionally by increasing the temperature or entropy of the system [58]. Therefore, the gel formation of IBU-based ISGs in the periodontal pocket at body temperature might be slightly more difficult than the in vitro gel formation experiment; nevertheless, the small amount of this dosage form of injection could be surrounded or immersed in crevicular fluid in the periodontal pocket for phase transformation induction.

#### 2.1.5. In Situ Gel Transformation

**Figure 1:**
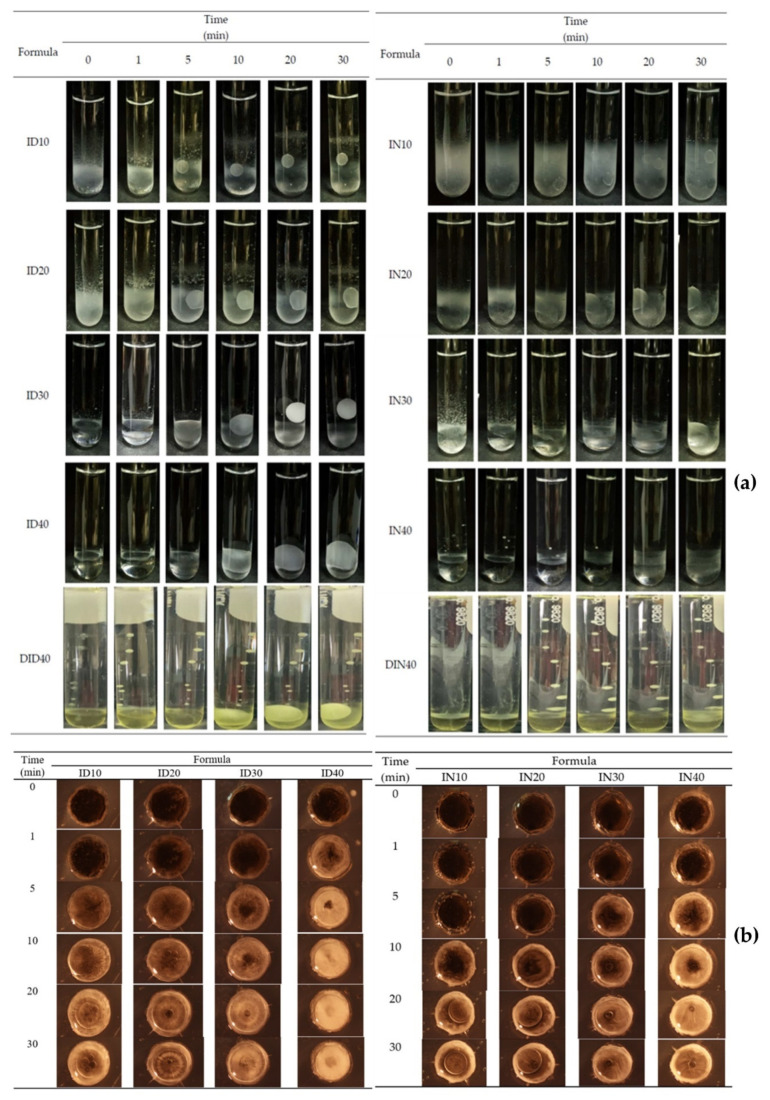
In vitro matrix formation behavior of IBU-based ISG and DH-loaded IBU-based ISG in PBS (**a**) and cross-sectional view of gel formation of IBU-loaded ISM systems using DMSO and NMP as the solvents in the agarose well under a stereo microscope at a magnification of ×12 (**b**).

The gel formation of IBU-based ISMs was tracked in the mimetic periodontal fluid. They transformed from solution into gel over time in the form of an opaque mass after injection into the PBS pH 6.8 buffer at the bottom of the test tube, as depicted in Figure 1a. Only ID30 and ID40 were transformed into gels completely within 30 min. At the initial stage, the injected solution formulation was liquid, accumulated at the bottom of the test tube, and seemed immiscible with PBS. As time passed, the IBU-loaded ISGs gradually transformed into spherical, light, soft gels that floated up from the bottom because the density of the formulation was reduced after the organic solvent diffused outward. For the NMP series, their ISGs presented as a turbid emulsion that dispersed around the bottom of the test tube and did not accumulate in a singular unit mass as observed for the DMSO series. The rather slow transformation was due to high affinity between IBU and these two solvents [29,56,57]. The transformation of DH-loaded IBU-based ISGs was slower than DH-free formulations. Therefore, DH altered both the appearance and transformation rate of the gel. The appearance of the gel changed from its original color to yellowish, which is the color of DH, and the gel transformation process exhibited a slower rate than the ID40 formulation. Even though the water tolerance of ISGs was reduced by the addition of DH, the rate of gel formation seemed to be unrelated to water tolerance because DH increased the viscosity of ISGs and retarded solvent exchange and removal from the formulation. To obtain a clear understanding of phase transition, the morphological change of the formulation after filling the formulations into the agarose well was observed under a stereoscope, as shown in Figure 1b. The opaque ring expansion into the inner agarose well of the formulation was evident with time, particularly for high IBU loading formulas, such as ID30, IN30, and IN40. Formulations using DMSO as a solvent with high IBU loading exhibited more rapid phase transformation than that using NMP because of the higher polarity of DMSO provoking more miscibility between this solvent and the water phase, resulting in a rapid solvent exchange behavior and, thereafter, notable phase separation of IBU [28,57]. This result corresponded with water tolerance, as previously mentioned.

**Figure 2:**
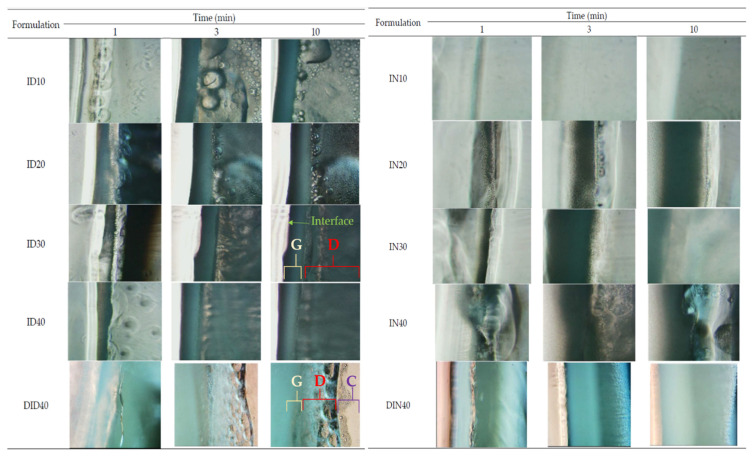
Matrix formation at the interface of the aqueous phase and ISG systems during the initial period (i.e., 1, 3, and 10 min) at a magnification of ×100 under an inverted microscope. Abbreviations: G = gel layers, D = droplet fluctuation layers, C = clear ISG layers.

Focusing on the microscopic aspect of gel formation around the interface among ISGs and aqueous solutions, the experiment was performed by dropping the formulation beside the PBS pH 6.8 agarose gels under an inverted microscope and the results are presented in Figure 2. This experimental design was conducted to investigate the phenomenon at the boundary of the formulation, which faced the target site containing the biological fluid [27,28]. Notably, during the gel formation process of all formulations, three different layers—namely, the gel formation layer (G layer), the droplet fluctuation layer (D layer), and the clear ISG solution layer (C layer)—were generated, as shown in Figure 2. The initial phase of gel transformation presented only a thin D layer between the interface and the C layer. For the D layer, the movement of droplets occurred from the interface part into the C layer; thus, this phenomenon was similar to emulsion droplet movement. As time passed, the D layer expanded to the C layer, and the G layer was generated markedly around the interface. The layers presented as a birefringent denser liquid layer that turned into a small crystal-containing layer. Empirically, the energy that exists at the interface between any unmixable object could induce nucleation; thereafter, crystallization occurs through a thermodynamic driving force [26,27]. Matrix growth at the interface of borneol-based in situ forming matrices and agarose rim occurred over time, and its formation became more rapid with the increase in borneol concentration in DMSO [15]. By comparison, the DMSO series ISGs presented more droplets with more fluctuation in droplet size than the NMP series. At higher concentrations of IBU, the droplet amount and its movement in the D layer increased. The G and D layers were also thicker than the low-IBU concentration formulation. The DH-loaded formulation presented a slower movement of droplets than the DH-free formulation. Moreover, the change of the D and G layers was slower; therefore, the higher viscosity of the formulation because of the addition of DH interrupted solvent removal and retarded the IBU matrix formation process.

#### 2.1.6. Injectability Properties

The injectable formulation should be controlled in its injection force and the amount of dosage form sufficient to eradicate the pathogen microbes. Moreover, these formulations can transform themselves from a liquid solution into a gel state; thus, ISG formulations should be injected rapidly to ensure homogeneous matrix formation [16,47]. The work and force of injection were apparently increased with the increase in IBU content in the formulation and quite higher in the NMP series than in the DMSO series (Table 3), corresponding well with the viscosity values shown in Table 1. DH-loaded IBU-based ISGs exhibited the same trend, in which DIN40 exhibited slightly higher force and work of injection than DID40. The force and work of injection of DIN40 were 2.543 ± 0.022 N and 48.357 ± 0.560 N/mm, respectively, and those of DID40 were 2.365 ± 0.013 N and 43.242 ± 0.200 N/mm, respectively. The force and work of injection of these two solvents were both quite significantly lower than the formulations (*p* < 0.05). The force of injection of DH-loaded IBU-based ISGs was significantly lower than the acceptable level of force for injectable formulation (<50 N), indicating that it was conveniently applied to the administration site [59]. From previous polymer-based ISG studies, the matrix/gel-forming materials, i.e., ethyl cellulose, Eudragit RS, and bleach shellac [23,25], exhibited markedly higher viscosity and force of infection than the IBU-based ISGs. Therefore, the low viscosity and ease of injectability could diminish the pain from the injection of the formulation at the target site.

#### 2.1.7. Mechanical Properties

The mechanical properties of transformed gels in simulated periodontal pockets were tested using the texture analyzer. The transformed gels of ISGs should adhere to the pocket and resisted the jaw’s motion to retain its shape to fit the periodontal pocket [60]. The mechanical properties were calculated based on the maximum and remaining force to determine the elasticity or plasticity of gels. The adhesive force and mechanical properties of the transformed gels of IBU-based and DH-loaded IBU-based ISGs are presented in Table 3. The addition of DH increased the hardness of gels as revealed by the maximum compression forces of ID40 and DID40 (i.e., 0.736 ± 0.001 and 1.678 ± 0.161 N, respectively). Moreover, DIN40 presented higher hardness than DID40 as indicated by the maximum compression force. DIN40 also exhibited higher remaining force and mechanical properties with similar adhesion force to DID40. This result corresponded with the previous report of bleached shellac-based ISG, with the mechanical hardness of its transformed matrix gel prepared with three solvents ordered as 2-pyrrolidone > NMP > DMSO, which was influenced by the phase separation rate [53]. The obtained transformed bleached shellac systems prepared with NMP and DMSO were more likely plastic or able to adapt their geometry to dynamic changes, whereas those prepared with PYR were elastic [53]. During the texture analysis, the probe pulled back all of the fabricated formulations presenting a low adhesive strength property against the texture analyzer probe and all obtained gels that are still attached to the agarose basements with different values (Table 3). This adhesive quality between the transformed ISG and the target site was designed to prevent the undesirable slipping out of the dosage form from the periodontal pocket [46,53].

#### 2.1.8. In Vitro Drug Release

**Figure 3:**
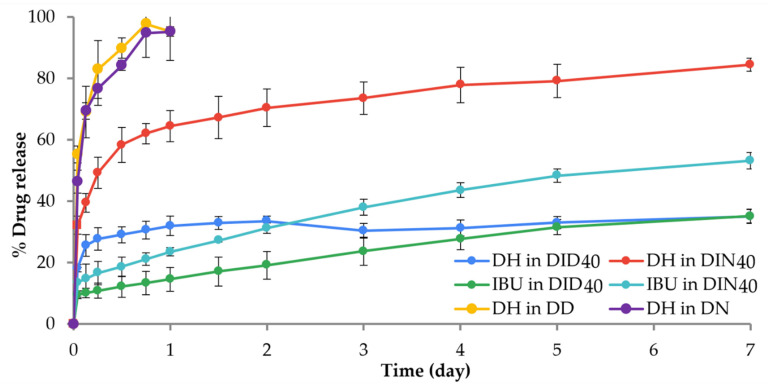
Comparison of the release profiles of DH and IBU from DH-loaded 40% IBU ISG systems (*n* = 3).

**Figure 4:**
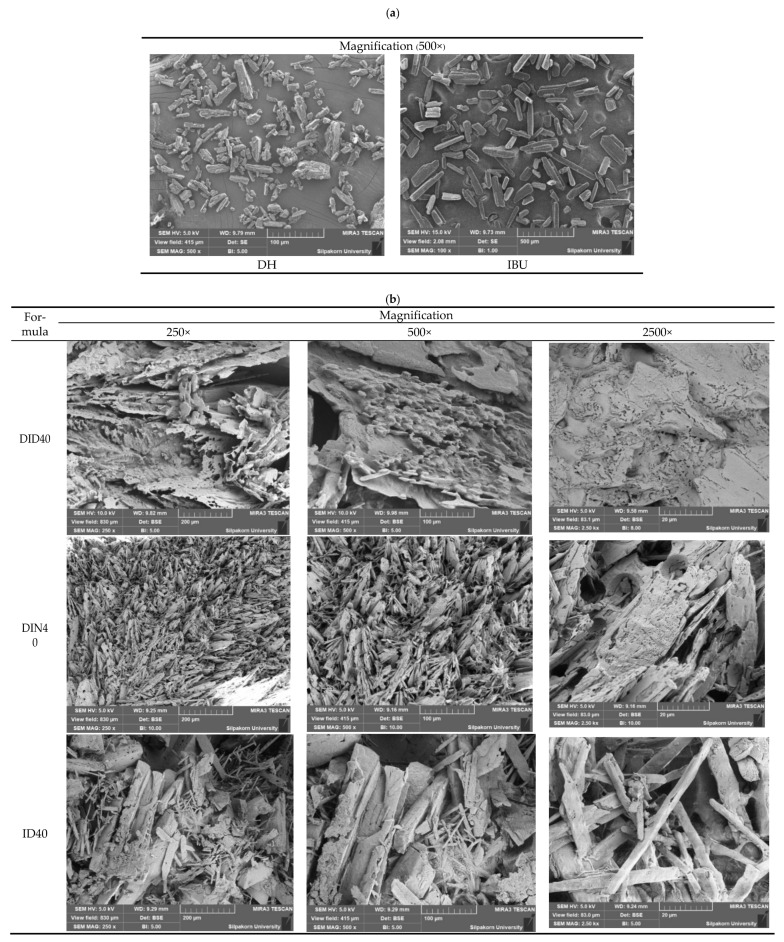
SEM photographs of DH and IBU powders (**a**) and remnants from DH-loaded IBU ISM systems using DMSO and NMP as the solvents and IBU in DMSO (ID40) after drug release test in PBS pH 6.8 for 7 days (**b**).

DH-loaded IBU-based ISGs were designed as a drug delivery system for the treatment of periodontitis under the concept of “using the active chemical as excipient with dual drug release” [34,40,41]. This concept applies the physical and chemical properties of active ingredients to assist the preparation or enhance the efficacy of the main active pharmaceutical ingredient. IBU-based ISMs using these solvents have been identified as suitable drug delivery systems because of their low viscosity, thereby facilitating injectability and safety in humans given the low toxicity of DMSO (LD_50_ = 2.5–8.9 g/kg) [61] and NMP (oral and dermal LD_50_ in rodents of 3600–7700 mg/kg) [62]. The IBU concentration for ISG drug delivery system fabrication was 40% *w*/*w* because this concentration was the highest concentration of IBU that was able to load 5% *w*/*w* DH. The slow transformation rate into gel was evident for ISG containing IBU less than 40% *w*/*w*, in which these systems might leak out from the periodontal pocket before matrix formation. Hence, 40% *w*/*w* IBU was selected for loading DH, such as DID40 and DIN40 formulations. IBU was used as a matrix-forming material for controlling DH release to reduce administration frequency with the anti-inflammatory activity of IBU for relief of the inflammation of gingival tissue or gum. In vitro drug release study of DH-loaded IBU-based ISGs was performed by the cup method to simulate the environment of the periodontal pocket [14,15]. Moreover, many research works have applied the cup method to investigate drug release. DH was liberated from various ISGs comprising 40% Eudragit RS, 30% bleached shellac, 40% beta-cyclodextrin, and 20% Ethocel in 16 h, 2 days, 7 days, and 2.5 days, respectively, using this technique [20,21,25,63]. The control groups, such as DD and DN, were released suddenly into PBS pH 6.8 solution (Figure 3). DID40 and DIN40 solutions in the cup were transformed into turbid gels completely within 15 min and 2 h, respectively. The final remnant of DID40 and DIN40 ISGs was a solid matrix and presented as a complete transformation within 6 h and 1 day, respectively. Atridox^®^ containing DH using 33.03% poly(d,l-lactide) as a polymer controls the drug release for 7 days after the periodontal pocket is cleaned with an irrigating solution [17,18]. The flushing of an irrigating solution, such as chlorhexidine solution, reduced the plaque bacteria before administration with the prolonged DH delivery system [64]. Hence, DH-loaded IBU-based ISG formulations in this study should control DH release for not less than 7 days. Considering the release profile shown in Figure 3, DH dissolved in organic solvent without IBU (gel-forming agent) as DD and DN exhibited burst release to 100% within the first day. However, DID40 and DIN40 exhibited burst release at the initial phase or within 3 and 18 h, respectively. After the initial phase, the DH release rate decreased and turned to controlled release over time. Their DH release behavior is related to the state of the gels because the solid mass of the obtained hydrophobic IBU matrix could obstruct DH release more than the liquid and gel states [65]. Moreover, the efficiency of controlling DH release from the system corresponded well with the topography of the solid matrix of IBU from the SEM result (Figure 4), because DID40 had multiple layers of tortuously organized sheet structure with a small porous surface to impede drug liberation similar to drug diffusion through a matrix of saturated fatty acid-based ISG as previously reported [26,27,28]. Meanwhile, DIN40 showed the complexation of needle crystals with spherical holes and more porosity as multichannel (Figure 4) for drug diffusion outward into the medium. Thus, these IBU matrix structures enhanced the efficacy of DID40 to entrap DH in the system and prolonged DH release superior to that of DIN40. The percentage of DH release of DID40 and DIN40 on the seventh day was 38.72 ± 0.19% and 84.49 ± 2.08%, respectively. Nevertheless, both of these ISGs exhibited efficient controlled DH release with sustainable local drug release in the periodontal pocket for enhanced patient compliance because of reduced medication frequency [11].

For fitting different mathematical models to obtained drug release profiles, DDSolver^®^ software applies the nonlinear least square curve-fitting technique, which determines the parameter values by minimizing the sum of squares (SS) or the weighted SS [66]. The conventional or modified forms of the utilized mathematical models were chosen from the model library collected based on the established models in the previous studies [67]. The estimated parameters from the DH release profile were fitted to the obtained release profiles of DH with zero-order, first-order, Higuchi’s, Korsmeyer–Peppas’s, Hixon’s, Hofenberg’s, and Peppas–Sahlin’s equations, as presented in Table 4. The DH release profile of DID40 fits quite well with Korsmeyer–Peppas’s model (*R*^2^ = 0.9345, AIC = 57.4259, MSC = 1.2889) and Peppas–Sahlin’s model (*R*^2^ =0.9871, AIC = 13.5315, MSC = 43.4335). Therefore, DH was released from the system proportionally to the exponent of time and the release mechanism followed the Fickian diffusion mechanism dominantly that occurred through the usual molecular diffusion of the drug because of a chemical potential gradient. The dominant drug release mechanism was determined to be the Fickian diffusion mechanism because the diffusion exponent (*n* value in Korsmeyer–Peppas’s model (*n* = 0.0861) and *m* value in Peppas–Sahlin’s model (*m* = 0.2636)) was less than 0.45, and when comparing the *k*_1_ and *k*_2_ values in Peppas–Sahlin’s model, *k*_1_ was superior to *k*_2_ (*k*_1_ = 53.2199 and *k*_2_ = −22.6506); thus, the ratio of the Fickian diffusion mechanism was superior to the Case II relaxation mechanism [68,69]. DIN40 also fits Korsmeyer–Peppas’s model (*R*^2^ = 0.9796, AIC = 40.7243, MSC = 2.8788, and *n* = 0.1984) and Peppas–Sahlin’s model (*R*^2^ = 0.9857, AIC = 60.2321, MSC = 3.4269, *m* = 0.2402, *k*_1_ = 75.0866, and *k*^2^ = −11.5256) with the dominant drug release mechanism being the Fickian diffusion mechanism, similar to the DID40 formulation.

Typically, the pharmacological treatment of periodontitis involves the treatment of infection and inflammation. Hence, the IBU release behavior of ISGs should be monitored, as well as DH. The IBU release profiles are shown in Figure 3. Burst release of IBU occurred at the initial phase and ended within 1 h. Afterward, the release behavior changed to sustained release. IBU was released from ISGs at 35.17 ± 2.23% for DID40 and 53.19 ± 2.66% for DIN40 on the seventh day. An in situ forming implant fabricated using poly(d,l-lactic-co-glycolic acid) as polymer released both drugs (i.e., IBU and chlorhexidine dihydrochloride) in a controlled manner, in which the release rate of chlorhexidine dihydrochloride was higher during the first 5 days and then slowed down [41]. DID40 retarded IBU release from the system more efficiently than DIN40 and corresponded with that of the DH release behavior because the duration of the transformation from liquid state into gel state for DIN40 was noticeably longer than that for DID40. At the initial phase of ISG transformation, ISGs of DIN40 were in the liquid and gel states in this phase that IBU freely moved and easily diffused outward from the system and expressed a burst release behavior [65]. Thus, DIN40 presented apparent burst release behavior. For the later phase of drug release from the solid state of the IBU matrix, DIN40 also exhibited faster IBU release because its topographical surface was rougher and more porous with a larger water contact area than DIN40 [70]. By comparison, hydrophilic drugs, such as DH, exhibited a high initial burst release and less sustained release (Figure 3) because of their miscibility with the aqueous phase, whilst hydrophobic drugs, such as IBU, exhibited lower initial burst release and more sustained release because of their high affinity with the solvent. The apparent high loading of these two active compounds in the developed ISG dosage forms ensures efficient therapeutic periodontitis treatment [20,21,25]. The release profile of IBU also fits Korsmeyer–Peppas’s model (DID40: *R*^2^ = 0.9433, AIC = 53.1028, MSC = 2.7078, and *n* = 0.3875; DIN40: *R*^2^ = 0.8885, AIC = 60.2584, MSC = 3.0058 and *n* = 0.364) and Peppas–Sahlin’s model (DID40: *R*^2^ = 0.9474, AIC = 52.4558, MSC = 2.7576, *m* = 0.282, *k*_1_ = 10.363, and *k*_2_ = 5.713; DIN40: *R*^2^ = 0.9665, AIC = 60.0905, MSC = 3.0187, *m* = 0.3400, *k*_1_ = 20.2566, and *k*_2_ = 5.3191) (Table 5), similar to those of DH from IBU matrices. Moreover, the Fickian release mechanism was presented as the major mechanism in both DID40 and DIN40 similar to the DH release behavior. Thus, DH and IBU release from both DID40 and DIN40 were influenced by their concentration gradients through the IBU matrices.

#### 2.1.9. Surface Topography

The surface topography of intact drug powders and water-attached surfaces of dried gel remnants was observed using the SEM technique, as presented in Figure 4. The intact DH micrograph (Figure 4a, left) indicated the different sizes of crystalline rod-shaped powders with more aggregation than that of IBU (Figure 4a, right). The topography of intact IBU was a crystalline rhombic prism with various sizes and smooth surfaces. 

**Figure 5:**
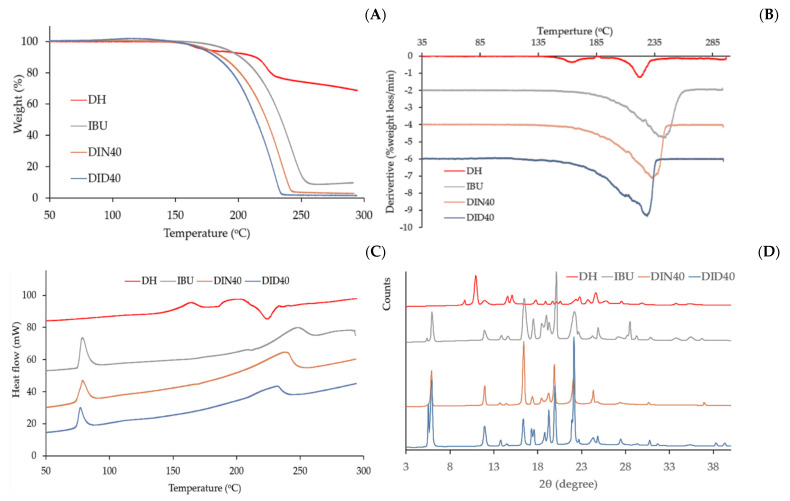
TGA (**A**), dTGA (**B**), and DSC (**C**) thermograms, and XRD diffractograms (**D**) of DH, IBU, and remnants from DH-loaded IBU-based ISG formulations after drug release.

DH-loaded ISG (DID40) remnant presented a rough homogeneous sheet with multiple layers of tortuously organized sheet structure with many small pores on the structure as observed at a high magnification of ×2500. The surface topology of ID40 dried remnant is composed of a network of large IBU crystals connected, as shown in Figure 5B, indicating IBU recrystallization with a rather slow crystallization rate. Therefore, the deconstruction force of DID40 from the mechanical study was somewhat stronger than that of ID40 against compression force. This result corresponded decently with the continuous/denser topography and the more compact component of the obtained mass of DID40 than ID40. Thus, DID40 tolerated the compression force more than ID40. 

However, the dried remnant of DIN40 exhibited compaction of needle crystals with many small circular holes around the surface. This apparent high porosity of DIN40 indicated the high rate of solvent exchange during its phase transformation. These topographical results from SEM explicated the DH and IBU release behavior as previously mentioned, in which the rates of liquid and drug diffusion were the limiting step of drug release through the different porous insoluble IBU matrices [71,72]. SEM of the dried matrix of vancomycin hydrochloride-loaded lauric acid-based ISG presented a needle-shaped crystal agglomerate with a continuously intricate matrix, whereas palmitic acid dried matrix of this dosage form showed an enclosed sheath crystal with a large and simple aperture, confirmed the low tortuosity which resulted in a burst drug release and low sustainable drug liberation [26,27]. Thus, the morphology of the transformed matrix of ISG notably influenced the drug release behavior. The aforementioned morphologically transformed matrices from saturated fatty acids and IBU were notably different from polymeric material-based ISG. The fibrous and scaffold characteristics were typically found for polymer-based ISG after solvent removal because of solvent leaching out from the gel structure promoting various pore inner remnant structures [21,23,24,50,73]. The SEM micrograph of the remnant from DH-loaded Eudragit RS ISG contained many spherical particles connected and formed into the continuous phase in which the size of the void inside the remnant structure decreased with the increase in polymer concentration [20,74]. 

This characteristic indicated the involvement of both the diffusion of the drug and the dissolution of the Eudragit RS network in controlling drug release. Therefore, the type of matrix-forming agent prominently influences the topography of the transformed structure of ISG systems after solvent removal and thereafter also affected the drug release characteristics.

#### 2.1.10. Differential Scanning Calorimeter (DSC), Thermogravimetric Analysis (TGA), Powder X-ray Diffraction (PXRD), and FOURIER Transform Infrared Spectroscopy (FTIR)

The decomposition pattern of intact components in ISGs and dried remnants are shown in the TGA and dTGA thermograms depicted in Figure 5A,B, respectively, with the patterns of their peak position listed in Table 6. The degradation temperature of intact DH exhibited two peaks at 160.72 °C and 222.07 °C, whereas the intact IBU presented a decomposition temperature at 232.25 °C. The dried remnants of both DID40 and DIN40 presented similar degradation patterns with slightly lower temperatures than intact IBU. This evidence indicated that these remnants had only IBU left in gel debris and presented less crystallinity than intact IBU. From the DSC thermogram (Figure 5C), the endothermic peaks of intact IBU appeared at 78 °C and 247 °C. The peak at 78 °C was the melting point of IBU [75] and the second peak indicated the decomposition of IBU, whereas DH showed two endothermic peaks at 163 °C and an endothermic peak for its melting point at 202 °C [76]. The DID40 and DIN40 remnants after the dissolution test showed quite similar peak patterns as intact IBU. The first peak of DID40 and DIN40 was the melting point of IBU that presented a similar pattern of endothermic temperature, whereas the second peak of DID and DIN was related to the decomposition temperature of IBU, as presented in the TGA data (Table 6). Their endothermic peak patterns confirmed that only IBU remained in the dried remnants because the thermal properties of the DID and DIN formulations were similar to that of intact IBU.

**Figure 6:**
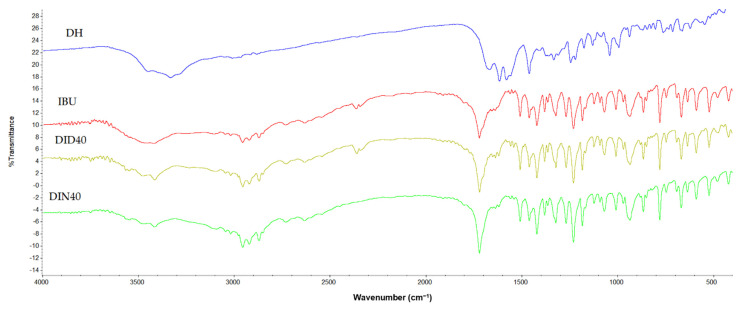
FTIR spectra of DH, IBU, and remnants from DH-loaded IBU-based ISG formulations after drug release.

To confirm the crystallinity of intact materials and dried remnants of ISGs, PXRD analysis was performed in an angular range of 3° to 40° 2*θ*, as shown in Figure 5D. The PXRD diffractogram of intact IBU and DH presented sharp peaks with different patterns, indicating that IBU and DH were crystalline materials. The characteristic peaks of intact IBU were observed at 5.9°, 11.9°, 16.4°, and 19.9° 2*θ* and those of DH were observed at 11° and 24.6° 2*θ*. The diffractogram of DID40 and DIN40 remnants also exhibited sharp peaks with similar peak positions to that of intact IBU. Meanwhile, dried gel remnants of DID40 and DIN40 had only characteristic IBU peaks. Furthermore, the intensity of their peaks presented a slight alteration compared with intact IBU. This change might correlate to the different recrystallization rates of IBU during its phase transformation when DMSO and NMP diffused outward, whereupon these solvents exhibited a different degree of affinity with the aqueous phase, as previously mentioned. The FTIR spectra of intact IBU showed intense and well-defined bands at 1720 cm^−1^ (carbonyl stretching of the isopropanoic acid group) and 3000 cm^−1^ (hydroxyl stretching), whereas DH showed a carbonyl band between 1714.717 cm^−1^ and 1649.138 cm^−1^ (Figure 6). Both DID40 and DIN40 debris showed similar patterns to that of intact IBU with no change in their peaks; thus, this result confirmed that no interaction between IBU with solvent and DH occurred [77]. The characterization studies of the dried remnants of DH-loaded IBU-based ISGs indicated that the crystalized IBU mass remained with no significant modification of its chemical structure and crystalline state.

### 2.2. Bioactivities Studies

#### 2.2.1. Antimicrobial Activities

**Figure 7:**
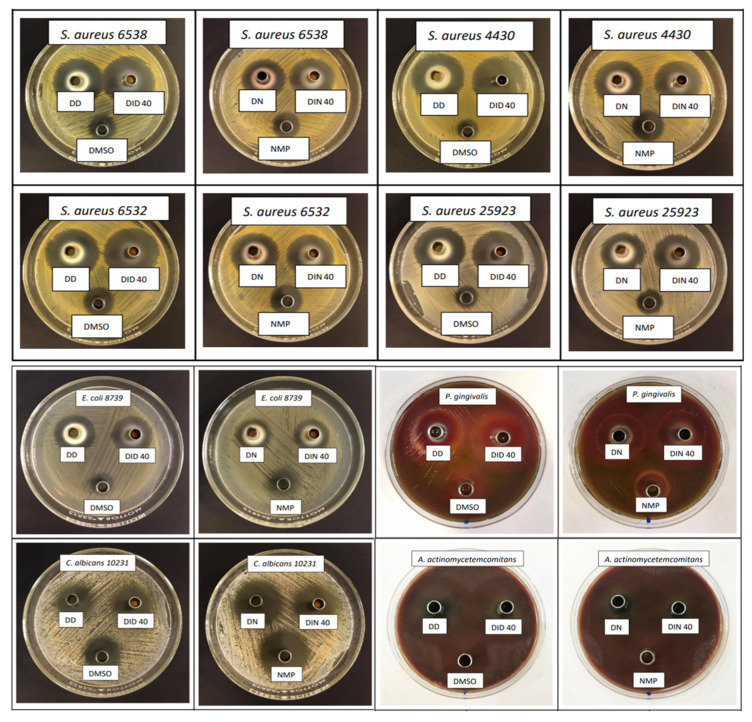
Inhibition zones of DH-loaded IBU-based ISG systems against *S. aureus* (ATCC 6538, 6532, and 25923), methicillin-resistant *S. aureus* (MRSA) (*S. aureus* ATCC 4430), *E. coli* ATCC 8739, *C. albicans* ATCC 10231, *P. gingivalis* ATCC 33277, and *A. actinomycetemcomitans* ATCC 29522 (*n* = 3).

The inhibition zone of the developed formula against *Staphylococcus aureus* (ATCC 6538, 6532, and 25923), methicillin-resistant *S. aureus* (MRSA; *S. aureus* ATCC 4430), *Escherichia coli* (ATCC 8739), *Candida albicans* (ATCC 10231), *P. gingivalis* (ATCC 33277), and *Aggregatibacter actinomycetemcomitans* (ATCC 29522) from the antimicrobial activity tests via the cup agar diffusion method is illustrated in Figure 7, and their measured inhibition zone diameters are shown in Table 7. These tested microbe species were selected because all of them are associated with periodontitis, particularly pathogens such as *P. gingivalis* and *A. actinomycetemcomitans*. DMSO and NMP are the organic solvents used as components in some pharmaceutical dosage forms because of their safety with low toxicity (LD_50_ of DMSO and NMP are 2.5–8.9 and 4.15 g/kg, respectively) [78]. In commercial products, 33.3% *w*/*w* NMP was used as a solvent of Atridox^®^ to dissolve the drug and poly(lactide) [79]. Furthermore, the US FDA has approved 50% *w*/*w* DMSO (RIMSO-50^®^) for the treatment of human interstitial or chronic cystitis, as well as various topical dosage forms containing DMSO for flap ischemia (60% DMSO), herpes zoster (5% idoxuridine in DMSO), and injection site extravasation (99% DMSO) treatment [27,80]. However, the safety data of various medical applications of DMSO, NMP, and the developed formulations need to be determined through clinical experiments. Both DH in organic solvent solutions (DD and DN) that served as positive control groups exhibited significantly larger clear zones against all of the tested bacteria strains than the organic solvents (*p* < 0.05), particularly DD, because DMSO showed less antibacterial activity. NMP exhibited notable antimicrobial activity against *C. albicans and A. actinomycetemcomitans*. This interesting antimicrobial activity of NMP has been reported previously [81]. Therefore, the use of NMP as a solvent could enhance the antimicrobial activities of ISG. By comparison, NMP and DN presented no significant difference in inhibition zone against *C. albicans*, because DH is not designed to inhibit this yeast and NMP efficiently inhibits fungus [20,81].

Notably, DH-loaded IBU-based ISGs exhibited a tendency toward more inhibition zones against nearly all microbial strains when compared with DH dissolved in organic solvents (Table 7). Therefore, IBU enhanced the antimicrobial activity of DH; nevertheless, the dominant antimicrobial activities were from broad-spectrum antibiotics, such as DH. The antimicrobial activities of IBU have been reported in some literature, including against Gram-positive bacteria, such as *Bacillus subtilis* and *S. aureus* with a minimum inhibitory concentration (MIC) of 1.25–2.5 mg/mL [82]. Its equipotent antibacterial activity against three *Gram*-negative human pathogens, namely, *E. coli*, *Salmonella typhi*, and *Enterobacter* sp., was reported with a MIC range of 2.5–5 mg/mL [83]. The IBU matrices that occurred after phase transformation did not markedly interfere with the inhibition efficacy, whereas the other matrices transformed from ISG prepared with natural resins [13,14,16], cholesterol [22], polymers [25], and saturated fatty acids [27] typically diminished the inhibition zone diameter. Therefore, IBU is a promising material providing a beneficial antimicrobial activity without lowering the inhibition efficacy when it was applied as the matrix-forming agent of ISG in addition to its capability to modulate drug release. The main pathogens of adult periodontitis are *P. gingivalis* and *A. actinomycetemcomitans*. However, the precise roles that such species play, singularly or in combination, in the pathogenesis of periodontal breakdown remain to be determined. In contrast to the majority of general infections, all of the suspected periodontal pathogens are indigenous to the oral flora, of which *C. albicans* causes refractory periodontitis [84]. *S. aureus* could be isolated from the periodontal pockets of patients with aggressive periodontitis and *E. coli* was considered a microorganism usually detected in patients with periodontitis [85]. From DH burst release on the first day of DH-loaded IBU-based ISGs, the drug amount at the initial stage was sufficient for inhibiting the growth of microbes. In patients with periodontitis, the volume of gingival crevicular fluid in the crevicular pocket ranges from 0.5 µL to 1.0 µL [86]. Once 0.3 g of DH-loaded IBU-based ISGs were injected into the crevicular pocket, the cumulative DH released from DID40 ISGs on the first and seventh days were 16,000 and 19,000 µg/mL, respectively, and those from DIN40 were 32,200 and 42,500 µg/mL, respectively, which are above the MIC of DH against *P. gingivalis* and *A. actinomycetemcomitans* (0.125 and 0.21 µg/mL, respectively) [87,88,89]. Thus, the localized DH-loaded IBU-based ISG should be able to effectively eradicate the pathogens and related microbes during periodontitis treatment.

#### 2.2.2. Anti-Inflammatory Activities

**Figure 8:**
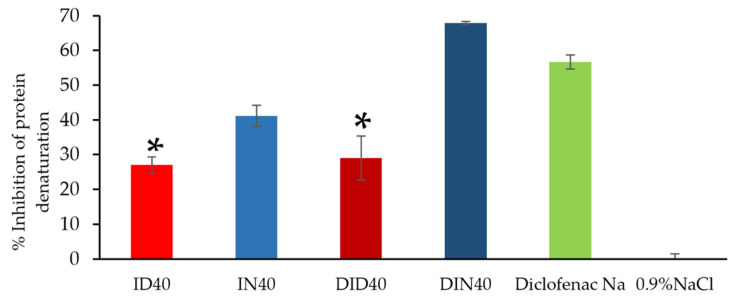
Percentage of inhibition of protein denaturation of DH-loaded IBU-based ISG (*n* = 3). The asterisk symbol indicates no significant difference (*p* ≥ 0.05) using one-way ANOVA followed by the LSD posthoc test.

Aside from the antimicrobial activities, the anti-inflammatory effect on periodontitis treatment has been the focus of considerable interest because inflammation is a complicated process that typically results in pain and involves changes to membranes and increases protein denaturation and vascular permeability [90]. The protein denaturation process is influenced by external factors, such as heat, organic solvents, and extreme changes in environmental conditions, such as pH or inorganic salt concentration changes [91]. The thermal inhibition of protein denaturation of egg albumin is one of the models used to screen the in vitro anti-inflammatory activity of a novel substance or formulation [90,91]. The percentage of inhibition of the protein denaturation effect on the release medium from the fabricated IBU-based ISGs and DH-loaded IBU-based ISGs on the first day is shown in Figure 8 using diclofenac sodium and 0.9% sodium chloride as the positive and negative controls, respectively. Only DIN40 exhibited higher anti-inflammatory activity than the positive control. The anti-inflammatory activity of DIN40 was superior to that of DID40 because IBU released from DIN40 was superior to that from DID40 (DIN40 was 23.58 ± 1.32 and DID40 was 14.58 ± 3.85). The percentage of inhibition of ID40 and DID40 was significantly less than that of DIN40 and the positive control (*p* < 0.05). Moreover, this experiment revealed the potential of DH to synergize the anti-inflammatory activity of ISGs by increasing the IBU activity in both the DMSO and NMP series. Doxycycline suppressed the inflammatory activity via the reduction of the production of the cytokines IL-1β, IL-6, TNF-α, and IFN-γ, as well as the chemokines MCP-1, MIP-1 α, and MIP-1β, in a dose-dependent manner [92]. By comparison, the activity of NMP series ISGs was superior to that of DMSO series ISGs. A previous study reported that intact NMP exhibited more anti-inflammatory activity than DMSO [93]. NMP, 2-pyrrolidone, and diclofenac sodium expressed concentration-dependent inhibition of heat-induced protein denaturation of egg albumin with 50% inhibitory concentration (IC_50_) values of 4.82% *v*/*v* (49.35 mg/mL), 4.83% *v*/*v* (52.29 mg/mL), and 736.50 µg/mL, respectively [93]. NMP has also been reported to decrease inflammation by activating Krüppel-like factor 2 [94] and suppressing nuclear factor kappa B signaling [95]. The potentially diminished inflammatory activity of the developed DH-loaded IBU-based ISGs is beneficial to periodontitis treatment.

## 3. Conclusions

IBU was designed as a matrix-forming agent for ISGs because of its water insolubility property. The addition of IBU lessens the density and surface tension of the IBU-based ISG solution. Although their water tolerance values decreased with the increase in IBU content and the addition of DH, the NMP series exhibited a higher water tolerance value than the DMSO series. When these IBU-based ISGs were exposed to the aqueous environment, the transformation from solution into a gel or matrix-like behavior occurred via the solvent exchange mechanism from the aqueous influx and solvent efflux. Phase separation was revealed by droplet fluctuation around the interface during the solvent exchange process. After this, the crystallization of IBU into the matrix was induced by diffusing the solvent and substituting water. The higher the concentration of IBU in the solvent was, the faster the gel transformation. DMSO promoted faster gel formation from solvent removal than NMP. These transformation results were influenced by the water tolerance property of the ISGs. The low contact angles of the DH-loaded IBU-based ISGs on an agarose gel surface that mimics the periodontal pocket surface indicated their good wettability and suitability for the periodontal pocket after injection. The TGA, DSC, and PXRD results of the dried gel remnants of ISGs after drug release signified no change in the IBU crystallinity and thermal properties after the phase transformation and drug release processes. Furthermore, the FTIR results confirmed that no chemical interaction among the ISG components occurred. By tortuously packing the obtained IBU matrix with small pores, the release behavior of both DH and IBU from ISGs was determined to be sustained over 7 days and obeyed the Fickian diffusion mechanism. The developed DID40 and DIN40 ISGs demonstrated efficient antimicrobial activities against *S. aureus* (ATCC 6538, 6532, and 25923), MRSA (*S. aureus* ATCC 4430), *E. coli* (ATCC 8739), *C. albicans* (ATCC 10231), *P. gingivalis* (ATCC 33277), and *A. actinomycetemcomitans* (ATCC 29522). IBU in the formulations enhanced the antimicrobial activity, whereas DH and NMP showed the potential synergistic effect on the anti-inflammatory activity of ISGs. Consequently, the DH-loaded solvent removal-induced IBU-based ISGs proposed in this study are a potentially effective bioactive drug delivery system for periodontitis treatment through periodontal pocket injection.

## 4. Materials and Methods

### 4.1. Materials

IBU (Lot No. 4000/1101/18/A-0150B, P.C. Drug Center Co., Ltd., Bangkok, Thailand) was used as the matrix-forming agent. DH (Lot No. 20071121, Huashu Pharmaceutical Corporation, Shijiazhuang, China) was used as the antimicrobial drug. NMP (≥99.5%, Lot No. 144560-118, QReC, Auckland, New Zealand) and DMSO (≥99.9%, Lot No. 1862992, Fisher Chemical, Horsham and Loughborough, UK) were used as the solvents. Potassium dihydrogen orthophosphate (Lot No. E23W60) and sodium hydroxide (Lot No. AF310204) from Ajax Finechem, New South Wales, Australia were used as the components of phosphate-buffered saline (PBS pH 6.8). Agarose (Lot No. H7014714, Vivantis, Selangor Darul Ehsan, Malaysia) was used to determine the gel formation behavior. *S. aureus* ATCC 6532, *S. aureus* ATCC 6538, *S. aureus* ATCC 25923*, MRSA* (*S. aureus ATCC 4430*), *E. coli* ATCC 8739, and *C. albicans* ATCC 10231 were obtained from the Ministry of Public Health, Mueang, Nonthaburi District, Thailand, whereas *P. gingivalis* ATCC 33277 and *A. actinomycetemcomitans* ATCC 29522 (MicroBiologics Inc., St. Cloud, MN, USA) were purchased from Thai Can Biotech Co., Ltd., Bangkok, Thailand. Tryptic soy agar (TSA) and tryptic soy broth (TSB) (Difco, Detroit, MI, USA) and sheep blood and chocolate agar (Department of Medical Science, Ministry of Public Health, Mueang, Nonthaburi District, Thailand) were used as media for antibacterial testing. Sabouraud dextrose agar (SDA) and Sabouraud dextrose broth (SDB) (Difco, Detroit, MI, USA) were employed for the antifungal test. Potassium bromide (KBr) (Spectrograde, Fisher Scientific UK Limited, Loughborough, UK) was used for the FTIR analysis.

### 4.2. Preparation of the ISG

IBU-based ISG systems were prepared by dissolving various concentrations of IBU (10%, 20%, 30%, and 40% *w*/*w*) in DMSO and NMP. DH concentration of 5% *w*/*w* was added to IBU (40% *w*/*w*) preparation. The mixtures were prepared by mixing using a magnetic stirrer at room temperature until completely dissolved. The components of the formulations are shown in Table 8.

### 4.3. Physicochemical Study

#### 4.3.1. pH, Density, and Viscosities

The pH values of all formulations were checked using a pH meter (Seven Compact, Mettler Toledo, East Bunker Ct Vernon Hills, IL, USA) in triplicate. The density was measured using a pycnometer (Densito 30 PX, Mettler Toled Ltd., PortableLab™, East Bunker Ct Vernon Hills, IL, USA) (*n* = 3). Viscosity and shear stress were measured using a viscometer (Brookfield Engineering Laboratories Inc., Middleborough, MA, USA) at 25 °C (*n* = 3).

#### 4.3.2. Surface Tension and Contact Angle

The contact angle values of the formulations on glass, paraffin, and agarose gel surfaces are measured using a goniometer (FTA 1000, First Ten Angstroms, Newark, CA, USA) with a pump-out rate of 1.9 µL/s. The contact angle at the time point of 5 sec is recorded (*n* = 3). Moreover, surface tension is checked from a pendant drop of a liquid ISG suspended in the air after injection using the instrument above with same pump-out rate (*n* = 3).

#### 4.3.3. Water Tolerance Measurement

This parameter signifies the durability of the ISG system to retain its clear solution against IBU precipitation once aqueous phase is included. This test was undertaken by adding 2.5 g of the ISG into a test tube. The20 µL deionized water was subsequently filled using a micropipette and mixing via a vortex mixer until it became turbid at 25 °C and 37 °C and calculated using Equation (1) (*n* = 3).

(1) $$\%\mathrm{water}\;\mathrm{tolerance}=\frac{\mathrm{water}\;\mathrm{amount}\left(g\right)}{\mathrm{sample}\;\mathrm{amount}\;\left(g\right)+\mathrm{water}\;\mathrm{amount}\;\left(g\right)}\times 100\%$$

#### 4.3.4. Gel Formation Study

This investigation was performed by injecting ISG through a 1-mL syringe through an 18-Guage needle into PBS (pH 6.8). The images indicate the gradual solution–gel transformation as a turbid layer occurred with time after the aqueous phase of surrounding agarose diffused to induce the phase separation of the formula. This transformation was recorded at different time intervals (i.e., 1, 5, 10, 15, and 30 min). The microscopic aspect of gel formation was investigated by injecting 150 µL samples into the agarose well (diameter of 7 mm). This well of agarose gel represents the crevicular pocket in humans. The change of this cross-sectional view of IBU matrix-like morphology is photographed under a stereo microscope (SZX10, Olympus Corp., Tokyo, Japan) at 0, 1, 5, 10, 20, and 30 min with SZX10 Series software.4.3.5. 

#### 4.3.5. Interfacial Phenomena of Formulation–Aqueous Phase

For the observation of the interface interaction, the agarose gel on glass slide was cut to the edge and ISG of 50 µL was dropped. The interface interaction was observed under an inverted fluorescent microscope (TE-2000U, Nikon, Kaw, Japan) by capturing the image at 1, 3, and 10 min.

#### 4.3.6. Injectability and Mechanical Properties

The DH-loaded fabricated ISGs were filled into a 1-mL syringe with 24-Gauge needle, which was clamped to a stainless steel stand and testing their injectability using. A texture analyzer (TAXT plus, Stable Micro Systems, Godalming, UK) in compression mode with the method as previously described [15] in triplicate.

The mechanical properties of the fabricated ISGs were measured using the above instrument. The agarose was made into a hole for adding formulations of 150 µL and kept for 72 h for completion of phase transformation. Subsequently, the probe of the instrument was driven down into the obtained gel at a rate of 0.5 mm/s. This position was held for 60 s, after which the probe was driven upward at a speed of 10 mm/s. The force measured at maximum probe penetration into the sample was called the maximum deformation force (the hardness), and the upward movement of the probe between the surface of the sample and the probe was recorded as adhesion force (*n* = 3).

#### 4.3.7. In Vitro Drug Release Studies

The in vitro drug release behavior of the ISG formulations was investigated using the porcelain cup method. First, 0.3 g of the samples of DH-loaded IBU-based ISG formulations and control formulations (DH dissolved in DMSO and NMP) were filled into the cup. Then, the filled cup was placed in a glass bottle containing 80 mL PBS (pH 6.8) and shaken using an incubator (Model NB-205, N-Biotek, Gyeonggi-do, Republic of Korea) at 37 °C with a rotational speed of 50 rpm. Finally, 3 mL of the release medium was sampled at specific time points and an equal volume of fresh PBS was replaced to maintain a sink condition.

The DH and IBU contents in the release medium at each specific time point were measured (*n* = 6) by high-performance liquid chromatography (Agilent 1260 Infinity, San Diego, CA, USA) at 264 nm using the C_18_ column (150 × 4.6 mm, 5 µm particle size; Dr. Maisch GmbH, Munich, Germany). The analyte was analyzed using gradient elution with the mobile phase that consisted of three solvents, i.e., 0.1% formic acid/acetonitrile/methanol with a flow rate of 1 mL/min. The gradient was applied according to the following program: 90:1:9 to 10:40:50 for 8 min, held at 50:20:30 for 5 min, and changed to 90:1:9 for 2 min. The intact DH and IBU were employed as the standard by dissolving in the release medium to obtain the calibration curve.

The drug release mechanisms were checked by fitting the obtained dissolution profiles with the following release mathematical models: zero-order, first-order, Higuchi, Korsmeyer–Peppas, Hixson, Hofenberg, and Sahlin–Peppas models. The DD-Solver software application, an add-in program for Microsoft Excel (Redmond, WA, USA) written in the Visual Basic application, was used to determine the release mechanism. The *n* value obtained by the Korsmeyer–Peppas equation was used to determine the drug release mechanism [66]. The coefficient of determination (*R*^2^), Akaike information criterion (AIC), and model selection criterion (MSC) were also calculated to indicate the degree of goodness from curve fitting.

#### 4.3.8. Scanning Electron Microscopy (SEM)

The topography of dried ISG systems was investigated using SEM (TESCAN MIRA3, Brno-Kohoutovice, Czech Republic) at an accelerating voltage of 15 kV. The remnant of each formulation after drug release test was washed with 200 mL distilled water five times before being immediately placed in an ultralow-temperature refrigerator (model: UF-V 500, BINDER GmbH, Tuttlingen, Germany) at −80 °C for 12 h and was freeze-dried using a freeze dryer (Triad™ Labconco, Kansas City, MO, USA). Then these dried specimens were kept in a desiccator for 72 h and coated with gold before examination via SEM at magnifications of ×250, ×500, and ×2500, compared with intact powder of DH and IBU at a magnification of ×500.

#### 4.3.9. Differential Scanning Calorimetry (DSC), Thermogravimetry (TGA), Powder X-ray Diffractometry (PXRD), and Fourier Transform Infrared (FTIR) Spectroscopy

Dried samples are first weighed at 2.5–4 mg and then placed in a non-hermetic aluminum pan. The pan is sealed, and the thermal property is determined by DSC and TGA. For DSC (DSC 8000, Perkin Elmer, Shelton, CT, USA), the samples are investigated under the heating rate of 10 °C/min and the heating range of 50 °C to 300 °C under 40 mL/min of nitrogen gas flow. For TGA (Pyris TGA, Perkin Elmer, Shelton, CT, USA), 5 mg of each sample is weighed, placed in a non-hermetic aluminum pan, and sealed. The heating rate is 10 °C/min with a heating range of 50 °C to 300 °C under 200 mL/min of nitrogen gas flow. Meanwhile, dTGA is calculated using the rate of percent weight change at each time. The crystalline property of the dried sample was observed using a powder X-ray diffractometer (XRD; Rigaku, MiniFlex II, Tokyo, Japan) in the range of 3° to 40° 2*θ*. The spectra of the samples were also recorded using an FTIR spectrophotometer (Nicolet 4700, Madison, WI, USA). A small amount of sample was blended with potassium bromide and compressed with a plunger and die to produce a pellet using a hydraulic press (Carver Press, Wabash, IA, USA) at a compression pressure of 4 tons. The fabricated pellet was mounted in an FTIR chamber. The percent transmittance of the sample was recorded. The spectra were acquired at a resolution of 2 cm^−1^ in the range of 400–4000 cm^−1^.

### 4.4. Bioactivity Studies

#### 4.4.1. Antimicrobial Activity

DH-loaded and drug-free ISG formulations were evaluated for their antimicrobial activities against standard microbes — (*S. aureus* DMST 6532, MRSA (*S. aureus* ATCC 4430), *S. aureus* ATCC 6532, and *S. aureus* ATCC 25923), *E. coli* ATCC 8739, *C. albicans* 10231, *P. gingivalis*, and *A. actinomycetemcomitans* — using the agar cup diffusion method. Bacteria inocula were incubated for 36 h in TSB, whereas the fungus was incubated in SDB which their turbidity of the broth suspensions was calibrated using the 0.5 McFarland standard. Then, the prepared broth suspensions of aerobe bacteria were swab spread on the TSA plates, whereas sheep blood and chocolate agar were used as the media for *P. gingivalis* and *A. actinomycetemcomitans*, respectively. The inoculum of calibrated *C. albicans* was swab spread on SDA. Sterilized cylindrical cups were carefully placed on the swabbed agar. A 200 µL aliquot of the prepared ISGs was filled to the cup and incubated at 37 °C for 24 h. An anaerobic incubator (Forma Anaerobic System, Thermo Scientific, Cincinnat, OH, USA) was employed to incubate two anaerobic bacteria at 37 °C for 72 h. The diameter of inhibition zone was measured using a ruler (*n* = 3).

#### 4.4.2. Anti-Inflammatory Study

The anti-inflammatory activity of the DH-loaded IBU-based ISG formulations was tested using the thermal inhibition of protein denaturation of egg albumin [63]. Briefly, a 5 mL solution containing 2.8 mL of PBS pH 6.4, 0.2 mL of egg albumin (from fresh hen’s egg), and 2 mL of a sample of the release medium from Day 1 of an in vitro drug release test (Section 4.3.7) was prepared. Double distilled water and diclofenac sodium were used as the negative and positive controls, respectively. The prepared solutions were first incubated at 37 °C for 15 min in the incubator and then heated in the water bath at 70 °C for 5 min. After cooling down to room temperature, the absorbance of the mixtures was measured at 660 nm using a UV–vis spectrophotometer (Hitachi U-2000, COAX Group Corporation Ltd., Bangkok, Thailand). The percentage inhibition of protein denaturation was calculated using Equation (2):

(2) $$\%\;\mathrm{inhibition}=\frac{\mathrm{absorbance}\;\mathrm{of}\;\mathrm{negative}\;\mathrm{control}-\mathrm{absorbance}\;\mathrm{of}\;\mathrm{sample}}{\mathrm{absorbance}\;\mathrm{of}\;\mathrm{negative}\;\mathrm{control}}\times 100$$

### 4.5. Statistical Analysis

Statistical significance was assessed by one-way analysis of variance (ANOVA) followed by the Tukey test. The analysis was conducted using SPSS for Windows (version 11.5). The significant level was set at *p* < 0.05.

## Figures and Tables

**Table 1:** Physical properties of ibuprofen (IBU) in NMP or DMSO and DH-loaded solvent removal-induced IBU-based ISG systems (mean + S.D.) (*n* = 3).

Formula	Density (g/cm^3^)	pH	Viscosity (cP)	Surface Tension (mN/m)	Contact Angle (Degree)		
					Glass Slide	Agarose Gel	Paraffin
ID10	1.0845 ± 0.0012	7.66 ± 0.06	4.02 ± 0.23	40.18 ± 0.41	18.75 ± 0.75	18.56 ± 3.62	55.17 ± 1.63
ID20	1.0758 ± 0.0020	6.99 ± 0.04	5.73 ± 0.37	36.32 ± 1.17	16.98 ± 3.86	16.10 ± 1.45	50.71 ± 2.49
ID30	1.0678 ± 0.006	6.38 ± 0.10	7.87 ± 0.17	35.94 ± 0.26	15.16 ± 1.19	16.50 ± 0.98	46.74 ± 4.26
ID40	1.0586 ± 0.0011	5.87 ± 0.04	12.28 ± 0.07 ^b^	35.10 ± 0.87	15.21 ± 0.25	15.98 ± 0.56	50.57 ± 4.76
IN10	1.0264 ± 0.0008	8.26 ± 0.19	3.80 ± 0.06	39.42 ± 0.19	12.43 ± 2.32	26.01 ± 0.64	50.04 ± 0.87
IN20	1.0260 ± 0.0003	7.50 ± 0.20	5.95 ± 0.45	38.39 ± 0.76	11.51 ± 1.04	27.95 ± 0.65	49.03 ± 1.74
IN30	1.0253 ± 0.0006	6.55 ± 0.08	8.17 ± 0.27	37.41 ± 0.69	16.63 ± 1.65	20.83 ± 0.61	47.42 ± 2.62
IN40	1.0245 ± 0.0009	5.79 ± 0.07	13.86 ± 0.10 ^b^	36.91 ± 0.24	15.68 ± 0.51	18.69 ± 0.60	51.30 ± 1.40
DID40	1.0690 ± 0.0000	4.93 ± 0.16	18.61 ± 0.19 ^c^	34.81 ± 1.88	15.42 ± 0.80	17.22 ± 0.82	53.60 ± 3.08
DIN40	1.0260 ± 0.0004	5.04 ± 0.08	20.82 ± 0.37 ^c^	36.73 ± 0.89	15.31 ± 1.31	20.05 ± 0.60	52.74 ± 0.71
NMP	1.0265 ± 0.0008	10.08 ± 0.58 ^a^	2.04 ± 0.13	39.31 ± 0.28	31.08 ± 0.40 ^d^	7.12 ± 1.51 ^e^	54.94 ± 1.31 ^f^
DMSO	1.0935 ± 0.0007	10.02 ± 0.11 ^a^	1.98 ± 0.09	43.95 ± 0.13	33.98 ± 1.25 ^d^	4.30 ± 1.19 ^e^	64.70 ± 0.81 ^f^

The superscripts a, d, and e in the columns represent a significant difference (*p* < 0.01) and the superscripts b, c, and f in the columns represent a significant difference (*p* < 0.05) within the tested formulations.

**Table 2:** Water tolerance value of IBU in NMP or DMSO and DH-loaded solvent removal-induced IBU-based ISG systems after titration with deionized water at temperatures of 25 °C and 37 °C (*n* = 3).

Formula	Deionized Water Amount (Mean ± S.D. (μL))		% Water Tolerance (Mean ± S.D.)	
	25 °C	37 °C	25 °C	37 °C
ID10	893.33 ± 9.43	1046.67 ± 24.94	26.27 ± 0.20	29.45 ± 0.49
ID20	726.67 ± 9.43	806.67 ± 24.94	22.47 ± 0.23	24.34 ± 0.57
ID30	593.33 ± 24.94	846.67 ± 24.94	19.13 ± 0.65	25.24 ± 0.55
ID40	480.00 ± 16.33	613.33 ± 24.94	16.06 ± 0.46	19.65 ± 0.64
IN10	1300.00 ± 28.28	1733.33 ± 24.94	34.14 ± 0.49	40.87 ± 0.35
IN20	1126.67 ± 9.43	1440.00 ± 28.28	31.00 ± 0.18	36.48 ± 0.46
IN30	766.67 ± 24.94	1133.33 ± 18.86	23.41 ± 0.58	31.13 ± 0.36
IN40	566.67 ± 18.86	766.67 ± 24.94	18.43 ± 0.50	23.41 ± 0.58
DID40	463.04 ± 10.27	604.38 ± 17.22	12.92 ± 1.04	17.02 ± 0.44
DIN40	550.41 ± 24.16	748.11 ± 20.16	15.03 ± 0.70	20.79 ± 0.41

**Table 3:** Summary of the injectability and mechanical parameters of DH, IBU, and remnants from DH-loaded IBU-based ISG formulations (*n* = 3; mean ± SD).

Formulation	Injectability Properties		Mechanical Properties			
	Injection Force (N)	AUC of Injection (N∙mm.)	Maximum Force (N)	Remaining Force (N)	Adhesion Force (N)	Mechanical Properties
DMSO	0.734 ± 0.047 ^a^	12.339 ± 0.375 ^c^	-	-	-	-
ID40	1.837± 0.010	33.882 ± 0.663	0.736 ± 0.001	0.275 ± 0.009	0.083 ± 0.002	0.374 ± 0.013
DID40	2.365 ± 0.013	43.242 ± 0.200	1.678 ± 0.161	0.792 ± 0.093	0.091 ± 0.001	0.471 ± 0.019
NMP	0.904 ± 0.171 ^b^	12.195 ± 0.369 ^d^	-	-	-	-
IN40 *	1.930 ± 0.053	36.291 ± 0.081	-	-	0.130 ± 0.003	-
DIN40	2.543 ± 0.022	48.357 ± 0.560	2.623 ± 0.079	1.477± 0.057	0.087 ± 0.009	0.563 ± 0.005

* IN40 did not transform into gels and (–) not determined, the superscripts a to d in the column represent a significant difference within the tested formulations (*p* < 0.05).

**Table 4:** Degree of goodness-of-fit and estimated parameters from the curve fittings of the DH release profiles of different ISMs in PBS pH 6.8 using the cup method (*n* = 3).

Formulation	Modelling	Criteria for Model Selection			Kinetic Parameters		
		*R* ^2^	AIC	MSC			
DD	Zero order	0.4415	32.6951	−1.0092	*k*_0_ = 396.66		
	First order	0.8925	39.1268	1.1125	*k*_1_ = 11.8593		
	Higuchi	0.7001	47.2998	−0.2496	*k*_H_ = 135.2473		
	Korsmeyer-Peppas	0.9845	23.8737	2.6192	*k*_KP_ = 105.0155	*n* = 0.1965	
	Hixson	0.7484	46.1530	−0.0585	*k*_HC_ = 1.9307		
	Hopfenberg	0.8656	41.1295	0.7787	*k*_HB_ = 0.0038	*n* = 4411.0392	
	Peppas-Sahlin	0.9905	13.9208	4.6098	*k*_1_ = 180.6271	*k*_2_ = 96.0516	*m* = 0.3160
DID40	Zero order	0.4050	24.4736	−1.0740	*k*_0_ = 136.33		
	First order	0.0682	33.3778	−1.5290	*k*_1_ = 1.0087		
	Higuchi	0.2194	61.0878	−1.2085	*k*_H_ = 31.3751		
	Korsmeyer-Peppas	0.9345	57.4259	1.2889	*k*_KP_ = 29.8363	*n* = 0.0861	
	Hixson	0.0042	33.7183	−1.5971	*k*_HC_ = 1.9307		
	Hopfenberg	0.2707	25.6048	−1.3568	*k*_HB_ = 0.0026	*n* = 734.1810	
	Peppas-Sahlin	0.9871	13.5315	3.4335	*k*_1_ = 53.9199	*k*_2_ = −22.6506	*m* = 0.2636
DN	Zero order	0.0736	43.8395	−1.4003	*k*_0_ = 218.1449		
	First order	0.8795	40.8805	0.8518	*k*_1_ = 9.4428		
	Higuchi	0.7863	36.4714	0.0734	*k*_H_ = 144.0345		
	Korsmeyer-Peppas	0.9789	24.8168	2.4043	*k*_KP_ = 101.2309n = 0.2166	*n* = 0.2166	
	Hixson	0.7086	55.0420	−0.0523	*k*_HC_ = 1.4516		
	Hopfenberg	0.8563	50.0978	0.6541	*k*_HB_ = 0.0030	*n* = 5010.6982	
	Peppas-Sahlin	0.9940	17.4275	3.8821	*k*_1_ = 224.8520	*k*_2_ = −157.0385	*m* = 0.4227
DIN40	Zero order	0.2189	38.5772	−1.1486	*k*_0_ = 144.8828		
	First order	0.2704	110.9836	−0.4770	*k*_1_ = 1.2346		
	Higuchi	0.1722	113.6096	−0.6790	*k*_H_ = 42.5461		
	Korsmeyer-Peppas	0.9796	40.7243	2.8788	*k*_KP_ = 63.3992	*n* = 0.1984	
	Hixson	0.3735	46.0956	−0.8243	*k*_HC_ = 0.5602		
	Hopfenberg	0.2039	112.9868	−0.6311	*k*_HB_ = 0.0004	*n* = 3235.7009	
	Peppas-Sahlin	0.9857	60.2321	3.4269	*k*_1_ = 75.0866	*k*_2_ = −11.5256	*m* = 0.2402

Remarks: *R*^2^ = coefficient of determination; *k* = rate constant; AIC = Akaike information criterion; MSC = Model selection criterion.

**Table 5:** Degree of goodness-of-fit and estimated parameters from the curve fittings of the IBU release profiles of different ISMs in PBS pH 6.8 using the cup method (*n* = 3).

Formulation	Modelling	Criteria for Model Selection			Kinetic Parameters		
		*R* ^2^	AIC	MSC			
DID40	Zero order	0.3656	86.5737	0.1331	*k*_0_ = 6.4001		
	First order	0.5031	83.3354	0.3822	*k*_1_ = 0.0818		
	Higuchi	0.8787	64.9331	1.7978	*k*_H_ = 14.0786		
	Korsmeyer-Peppas	0.9433	53.1028	2.7078	*k*_KP_ = 16.2576 k_KP_ = 29.8363	*n* = 0.3875	
	Hixson	0.4610	84.4003	0.3003	*k*_HC_ = 0.0251		
	Hopfenberg	0.4576	85.3405	0.2280	*k*_HB_ = 0.0001	*n* = 750.6823	
	Peppas-Sahlin	0.9474	52.4558	2.7576	*k*_1_ = 10.363	*k*_2_ = 5.713	*m* = 0.282
DIN40	Zero order	0.3184	99.2511	0.0064	*k*_0_ = 9.907		
	First order	0.5583	92.6213	0.5164	*k*_1_ = 0.1514		
	Higuchi	0.8885	70.2564	2.2367	*k*_H_ = 21.8944		
	Korsmeyer-Peppas	0.9637	60.2584	3.0058	*k*_KP_ = 25.729	*n* = 0.364	
	Hixson	0.4912	94.8484	0.3450	*k*_HC_ = 0.0440		
	Hopfenberg	0.5178	94.6278	0.3620	*k*_HB_ = 0.000	*n* = 1493.131	
	Peppas-Sahlin	0.9665	60.0905	3.0187	*k*_1_ = 20.2566	*k*_2_ = 5.3191	*m* = 0.3400

Remarks: *R*^2^ = coefficient of determination; *k* = rate constant; AIC = Akaike information criterion; MSC = model selection criterion.

**Table 6:** Summary of the thermal parameters of DH, IBU, and remnants from DH-loaded IBU-based ISG formulations after drug release from DSC and TGA.

Formulation	TGA Parameter						DSC Parameter					
	Onset Temp (°C)		Degradation Temp (°C)		End Temp (°C)		Onset Temp(°C)		*T* Peak (°C)		Enthalpy (∆H) (J/g)	
	Peak 1	Peak 2	Peak 1	Peak 2	Peak 1	Peak 2	Peak 1	Peak 2	Peak 1	Peak 2	Peak 1	Peak 2
DH	157.11	168.66	160.72	222.07	168.30	225.07	151.71	186.66	163.66	202.32	59.63	71.64
IBU	217.6	-	232.25	-	250.84	-	75.48	225.55	78.67	246.81	92.99	101.74
DIN	208.5	-	229.58	-	238.80	-	74.56	198.42	78.68	237.30	79.59	300.70
DID	199.15	-	228.69	-	232.80	-	74.15	200.9	77.01	231.29	72.10	207.95

**Table 7:** Clear zone diameters of DH-loaded IBU-based ISG systems against *S. aureus* (ATCC 6538, 6532, and 25923), methicillin-resistant *S. aureus* (MRSA) (*S. aureus* ATCC 4430), *E. coli* ATCC 8739, *C. albicans* ATCC 10231, *P*. *gingivalis* ATCC 33277, and *A*. *actinomycetemcomitans* ATCC 29522 (*n* = 3).

Formula	Clear Zone Diameter (mm.) (Mean ± S.D.)							
	*S. aureus* ATCC 6538	*S. aureus* ATCC 4430	*S. aureus* ATCC 6532	*S. aureus* ATCC 25923	*E. coli * ATCC 8739	*C. albicans* ATCC 10231	*P. gingivalis* ATCC 33277	*A. actinomycetemcomitans* ATCC 29522
NMP	15.7 ± 1.5	16.7 ± 0.6	16.0 ± 1.0	14.3 ± 0.6	15.3 ± 0.6	29.7 ± 0.6 ^j^	17.0 ± 1.0	47.7 ± 2.1 ^n^
DN	23.0 ± 1.0 ^a^	25.7 ± 0.6 ^c^	25.7 ± 0.6 ^d^	25.7 ± 0.6 ^f^	20.7 ± 1.2 ^h^	29.7 ± 1.2 ^j^	27.3 ± 1.5^l^	48.0 ± 2.0 ^n^
DIN 40	26. 0± 1.0 ^a^	27.3 ± 0.6 ^c^	27.3 ± 0.6 ^d^	27.0 ± 1.0 ^f^	21.3 ± 1.5 ^h^	27.7 ± 1.5 ^j^	27.0 ± 1.0 ^l^	47.0 ± 1.0 ^n^
DMSO	13.0 ± 1.0	12.7 ± 1.2	14.0 ± 1.0	12.3 ± 0.6	12.7 ± 0.6	21.0 ± 1.0 ^k^	14.3 ± 0.6	24.0 ± 1.0
DD	27.7 ± 0.6 ^b^	30.7 ± 0.6 ^d^	32.0 ± 1.0 ^e^	31.0 ± 1.0 ^g^	22.7 ± 0.6 ^i^	22.3 ± 1.2 ^k^	28.7 ± 0.6 ^m^	41.7 ± 1.5 ^o^
DID 40	29.7 ± 0.6 ^b^	31.0 ± 1.0 ^d^	32.3 ± 0.6 ^e^	31.7 ± 0.6 ^g^	23.3 ± 0.6 ^i^	22.7 ± 0.6^k^	30.7 ± 0.6 ^m^	40.0 ± 1.0 ^o^

The superscripts a–o indicate no significant difference (*p* < 0.05) using one-way ANOVA followed by the LSD posthoc test.

**Table 8:** Composition of DH-loaded IBU-based ISG formulations.

Formulation Code	IBU (% *w*/*w*)	DH (% *w*/*w*)	Organic Solvent (Adjust to 100% *w*/*w*)
ID10	10	-	DMSO
ID20	20	-	DMSO
ID30	30	-	DMSO
ID40	40	-	DMSO
IN10	10	-	NMP
IN20	20	-	NMP
IN30	30	-	NMP
IN40	40	-	NMP
DID40	40	5	DMSO
DIN40	40	5	NMP

## Data Availability

The data presented in this study are available on request from the corresponding author.
